# From Waste to Worth: Using Fermented Orange Pomace in Sustainable Feed Production

**DOI:** 10.3390/plants13223191

**Published:** 2024-11-13

**Authors:** Tamer Elsakhawy, Tarek Alshaal, Ammar Elakhdar, Ibrahim El-Akhdar

**Affiliations:** 1 Agricultural Microbiology Research Department, Soils, Water and Environment Research Institute, Agriculture Research Center, Giza 12112, Egypt; drelsakhawyg@gmail.com (T.E.); dr.elakhdar@yahoo.com (I.E.-A.); 2 Department of Applied Plant Biology, Faculty of Agricultural and Food Sciences and Environmental Management, University of Debrecen, Böszörményi Str. 138, 4032 Debrecen, Hungary; 3 Soil and Water Science Department, Faculty of Agriculture, Kafrelsheikh University, Kafr El-Sheikh 33516, Egypt; 4 Field Crops Research Institute, Agriculture Research Center, Giza 12619, Egypt; a.elakhdar@kyudai.jp

**Keywords:** sustainable agriculture, orange pomace, fermentation, bio-fertilizer, barley biomass

## Abstract

Modern agriculture faces the dual challenge of producing environmentally friendly feed while minimizing chemical fertilizers and energy use. This study evaluates the use of orange pomace fermentation liquor (OPFL) as a bio-fertilizer to enhance the growth and nutritional content of sprouted barley for sustainable feed production. We conducted multiple assays to determine OPFL’s growth-promotion potential, including in vitro phosphate solubilization, indole-3-acetic acid (IAA) production, biofilm formation, and antimicrobial effects. Biosafety assays confirmed the absence of coliforms and hemolytic activity. Four barley varieties (Giza 2000, Giza 138, Giza 132, and Giza 126) were treated with OPFL in a hydroponic germination system, with significant improvements observed across several parameters. For example, in Giza 2000, chlorophyll content increased from 4.28 to 4.74, protein content rose from 12.15% to 22.07%, and plant height grew from 13.6 cm to 16.4 cm, representing increases of 10.7%, 81.6%, and 20.6%, respectively. Fresh biomass yield also saw a slight increase, though not statistically significant. This comprehensive evaluation suggests that OPFL is a sustainable alternative to chemical fertilizers, enhancing barley yield and quality in animal feed systems.

## 1. Introduction

The global agricultural sector faces increasing pressure to adopt sustainable practices that minimize environmental degradation while ensuring food security for a growing population. Traditional agricultural methods, particularly the use of chemical fertilizers, have been associated with several environmental issues, including soil degradation, water contamination, and the release of greenhouse gases. As a result, there is a growing need to explore eco-friendly alternatives that can maintain or even enhance crop productivity without further depleting natural resources [1,2]. Organic amendments, especially those derived from agricultural waste, have emerged as a promising solution to these challenges. By recycling organic materials, such as fruit and vegetable residues, these amendments provide a sustainable way to enrich soil and promote plant growth, while reducing the ecological footprint of farming practices. One such organic amendment is orange pomace fermentation liquor (OPFL), a byproduct of citrus processing that is rich in nutrients and microbial activity.

Citrus pomace, the solid residue left after extracting juice or other products from citrus fruits, includes seeds, pulp, and rinds. This byproduct is commonly discarded or used for low-value applications, such as animal feed or landfill disposal. However, citrus pomace is an underutilized resource, as it contains valuable organic compounds, including pectin, essential oils, sugars, and limonene [3]. Additionally, it is rich in bioactive compounds, such as flavonoids, polyphenols, and vitamins, which are known for their health-promoting properties. According to Widmer et al. [4], citrus pomace is composed of approximately 10% water, 14–25% pectin, 30–40% sugars, 0.4–1.6% fat, 13–17% cellulose and hemicellulose, and 0.5% micronutrients. Dry orange pomace also contains 5.9% protein, 3% ash, 1.52% total fat, and 89.50% carbohydrates [5]. The nutrient-rich composition of citrus pomace makes it an ideal candidate for fermentation processes that can transform it into a more valuable product for agricultural use.

Fermentation, an ancient process that uses microorganisms to break down organic matter, has long been recognized for its ability to improve the nutritional value of food and feed. In the case of citrus pomace, fermentation with lactic acid bacteria (LAB) can enhance its nutrient profile, making it a potent biostimulant for agricultural applications. LAB are known to convert sugars into organic acids, such as lactic acid, which lowers the pH and inhibits the growth of harmful pathogens. Additionally, LAB fermentation produces bioactive compounds, such as indole-3-acetic acid (IAA), gamma-aminobutyric acid, and exopolysaccharides, all of which can promote plant growth and improve soil health [6]. Spontaneous fermentation (SF), a process driven by naturally occurring LAB and other endogenous microorganisms, offers a sustainable and low-cost method of transforming citrus waste into a bio-fertilizer or feed additive. Aulitto et al. [7] noted that LAB strains isolated from citrus residues show strong probiotic potential and are effective in transforming citrus waste into high-quality fermented feed with extended shelf life.

The use of organic amendments, such as OPFL, aligns with the increasing demand for environmentally friendly agricultural inputs. LAB-based products have been successfully used in agriculture to improve soil fertility, control plant diseases, and enhance crop growth [8]. As bio-fertilizers, LAB can enhance nutrient availability by promoting the breakdown of organic matter and releasing nutrients, such as nitrogen and phosphorus, which are essential for plant growth [9]. Moreover, LAB act as biocontrol agents by producing antimicrobial compounds that suppress harmful pathogens, further improving plant health and resilience to stress [10]. The application of LAB-based organic amendments,, such as fermented orange pomace, represents a promising strategy for promoting sustainable farming practices while improving crop productivity.

Barley (*Hordeum vulgare* L.) is one of the most important cereal crops globally, cultivated for a variety of uses, including food, feed, and malt production. Due to its adaptability to a wide range of environmental conditions and its ability to grow in nutrient-poor soils, barley is a key crop in many regions, particularly in arid and semi-arid areas. However, improving its nutritional quality, especially its protein content, is crucial for enhancing its value as an animal feed and for meeting the needs of the brewing industry [1,11]. Traditional agricultural practices that rely on chemical fertilizers can enhance barley yield, but they often come with environmental costs. Organic amendments,, such as OPFL, offer a more sustainable alternative that can improve both the yield and nutritional quality of barley without the negative environmental impacts associated with synthetic fertilizers [12].

Sprouted barley, a method of producing forage grain without soil, has garnered interest due to its short growth period and high germination rate. The sprouting process activates enzymes that break down starch, protein, and lipids in the barley grain, resulting in a product with enhanced nutritional value that is easier for livestock to digest [13] (Tudor et al., 2004). Biofertilization with microbial inoculants,, such as LAB, during the sprouting process can further improve the biomass production and feed quality of sprouted barley [14]. Studies have demonstrated that microbial inoculants can enhance nutrient uptake, increase photosynthetic efficiency, and improve overall plant health, leading to higher yields and better-quality feed. Given the nutritional potential of OPFL and its ability to promote microbial activity in the soil, it could be an effective bio-fertilizer for sprouted barley production.

The primary objective of this study was to evaluate the potential of orange pomace fermentation liquor (OPFL) as a bio-fertilizer to enhance the growth, nutritional quality, and yield of sprouted barley varieties for sustainable feed production. To achieve this, specific microbiological assays were conducted to assess the bioactivity and safety of OPFL. These included tests for phosphate solubilization, indole-3-acetic acid (IAA) production, and biofilm formation, as well as biosafety assays for coliform detection and hemolytic activity. Additionally, the antimicrobial properties of OPFL were examined to determine its effectiveness against common plant pathogens. This comprehensive approach aimed to confirm OPFL’s viability as a safe and effective bio-fertilizer while assessing its potential to improve key physiological and morphological traits in barley. 

## 2. Materials and Methods

### 2.1. Preparation of Orange Pomace Fermentation Liquor 

**Figure 1:**
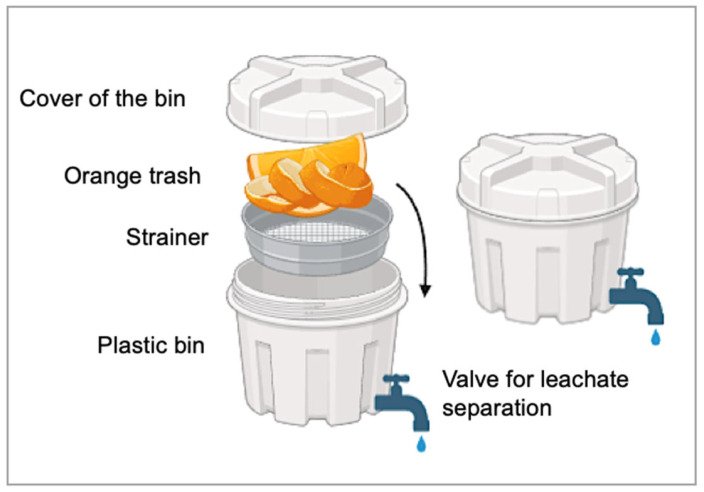
Custom-designed fermentation bin optimized for anaerobic fermentation of citrus pomace.

Orange pomace was collected from a nearby juice shop and immediately placed in a specially designed plastic bin. This bin was designed to facilitate successful anaerobic fermentation and allow for the easy extraction of the resulting fermentation liquor (Figure 1). The filled bin was incubated at 25 °C for 15 days with a relative humidity ranging between 50% and 60%. A valve was used to separate the fermentation liquor.

### 2.2. Assaying the Plant Growth Promotion Potential of OPFL

The plant growth promotion potential of OPFL was assayed by evaluating it possible in vitro phosphate solubilization capability, indole-3- acetic acid (IAA) and the ability to form biofilm. For the IAA formation assay, the OPFL was centrifuged at 5000 rpm, and supernatants were used for colorimetric measurement at 540 nm using UV/Visible Spectrophotometer (Jenway 6705; Cole-Parmer, Westwood Ave, Long Branch, NJ, USA) [15]. For phosphate solubilization, cultures were incubated on Pikovskaya agar with bromophenol blue [16]. Biofilm formation was measured using a 96-well microtiter plate assay with crystal violet staining [17]. 

### 2.3. Total Acidity 

To determine the total acidity of the fermented liquor, a titration method was employed. A known volume of the liquor was titrated with a standardized 0.1 M NaOH solution, using phenolphthalein as an indicator. The titration was performed until a persistent light pink color appeared, indicating the endpoint. The volume of NaOH used was recorded, and the total acidity was calculated as the equivalent concentration of acetic acid. The calculation was based on the formula:

$$\mathrm{Acidity}=\frac{V\times N\times MW}{V\;sample}$$

where V is the volume of NaOH used, N is the normality of the NaOH solution, MW is the molecular weight of acetic acid, and V sample is the volume of the sample. The total acidity was expressed as grams of acetic acid per liter of fermented liquor [18].

### 2.4. Biosafety Assay of Fermentation Product

#### 2.4.1. Detection of Coliform

The presence of coliform bacteria in the fermentation liquor was assessed by the Most Probable Number (MPN), following the method established by the American Public Health Association [19]. Samples were inoculated into MacConkey broth (typical composition (g/L): peptone from gelatine 20.0; lactose 10.0; dried ox bile 5.0; and bromocresol purple 0.01; pH 7.1–7.5; Merck KGaA, Darmstadt, Germany) and incubated at 35–37 °C for 48 h. Positive results were indicated by gas production and a color change due to lactose fermentation. To confirm the presence of coliforms, sub-culturing was performed on Eosin Methylene Blue (EMB) agar (typical composition (g/L): peptone 10, lactose 5; sucrose 5; dipotassium phosphate 2; agar 13.5; eosin Y 0.4; and methylene blue 0.065; pH to 7.2; Merck KGaA, Germany), followed by additional biochemical tests for further verification.

#### 2.4.2. Hemolytic Activity

To assess hemolytic activity, the fermentation liquor was centrifuged at 5000 rpm, and the resulting pellets were washed twice with phosphate buffer (pH 7.0) before being re-suspended in an equal volume of the same buffer. The suspension was then streaked onto blood agar (a nutrient-rich agar base supplemented with sterile blood, typically sheep or horse blood; Himedia Laboratories, India) plates and incubated under anaerobic conditions at 37 °C for 24 h. Hemolytic activity was indicated by the formation of a clear zone of erythrocyte lysis surrounding the colony [20].

### 2.5. Enumeration of Lactic Acid Bacteria in Fermentation Liquor 

Lactic acid bacteria (LAB) in the fermented pomace liquor were enumerated using MRS (typical composition (g/L): peptone 10; beef extract 10; yeast extract 4; glucose 20; sodium acetate trihydrate 5; tween-80 1; dipotassium hydrogen phosphate 2; and tri-ammonium citrate Tween^®^ 80 2; Merck KGaA, Germany) medium. The sample was diluted appropriately and inoculated into MRS agar plates. After incubation at 30 °C for 48 h, colonies were counted to determine the concentration of LAB in the fermentation liquor [21] De Man et al., (1960).

### 2.6. Antimicrobial Properties of Orange Pomace Fermentation Liquor

The antimicrobial activity of orange pomace fermentation liquor against *Pectobacterium carotovorum*—which was kindly obtained from the Plant Pathology Institute, Agricultural Research Center, Giza, Egypt—was assessed using the well diffusion technique. A bacterial suspension was prepared by growing the bacteria in nutrient broth (typical composition (g/L): D(+)-glucose 1; peptone 15; sodium chloride 6; and yeast extract 3; pH 7.5; Oxoid Ltd., Altrincham, Cheshire, UK) for 24 h, then uniformly spreading it on nutrient agar (typical composition (g/L): peptone 5; beef extract 3; NaCl 5; and agar 20; pH 7.4; Oxoid Ltd., UK) plates. Wells with 5 mm diameter were punched into the agar, and the fermentation liquor was added (50 µL), with sterile water serving as a control. The plates were incubated at 28–30 °C for 24–48 h, and the presence of inhibition zones after incubation indicates the antimicrobial effect. Experiments were conducted in triplicate. 

### 2.7. Barley Germination System 

Hydroponic cultivation and chemical analysis of sprouted grain were conducted according to established methods. The forage production unit utilized a hydroponic steel chamber with dimensions of 2.0 m in length, 2.0 m in height, and 1.0 m in width, designed to hold 21 steel perforated trays. Each tray, measuring 60 cm in length, 30 cm in width, and 5 cm in height, and the chamber conditions were maintained at a temperature range of 18–20 °C and a relative humidity of about 72% through air circulation. Fluorescent lighting providing around 1000 microwatt/cm^2^ for 9–12 h daily was used.

Barley seeds (*Hordeum vulgare* L.) of Giza2000, Giza 138, Giza 132, and Giza 126 varieties were obtained from the Barley Research Department, Crop Research Institute, Agricultural Research Center, cleaned, washed, and soaked in tap water for 24 h. They were then spread on the trays at a density of 0.5 kg per tray with a thickness of 2 cm. The experiment was divided into two groups; the first group was sprayed with 1:10 diluted OPFL, where each tray received 50 and the second group was sprayed with tap water and served as control, all the experiments proceeding in triplicate. After seven days of germination, the barley seedlings reached a height of 14–16 cm, forming a carpet-like appearance with dark green leaves and thick roots. The harvested grass carpets were tested for fresh weight, plant height, protein content, and chlorophyll content. The crude protein content was determined using the Kjeldahl method for total nitrogen with a conversion factor of 6.25 [22]. Concentrations of chlorophyll a, chlorophyll b, and carotenoids (mg/g FW) in the solution were determined using specific equations [23].

Chlorophyll a = 12.7 (A663) − 2.69 (A645)

Chlorophyll b = 25.8 (A645) − 4.68 (A663)

Carotenoids = (1000 (A470) − 2.27 (chl a) − 81.4 (chl b))/227

### 2.8. Statistical Analysis

Data were analyzed using COSTAT 6.303 software (CoHort Software, Monterey, CA, USA). A split–split plot design was employed to evaluate the effects of barley variety and inoculation with OPFL on the quality and biomass yield of sprouted barley. Analysis of variance (ANOVA) was conducted to determine the significance of treatment effects. Means were separated using the Least Significant Difference (LSD) test at the 0.01 level of probability.

## 3. Results

### 3.1. Characterization of the Fermentation Liquor

**Figure 2:**
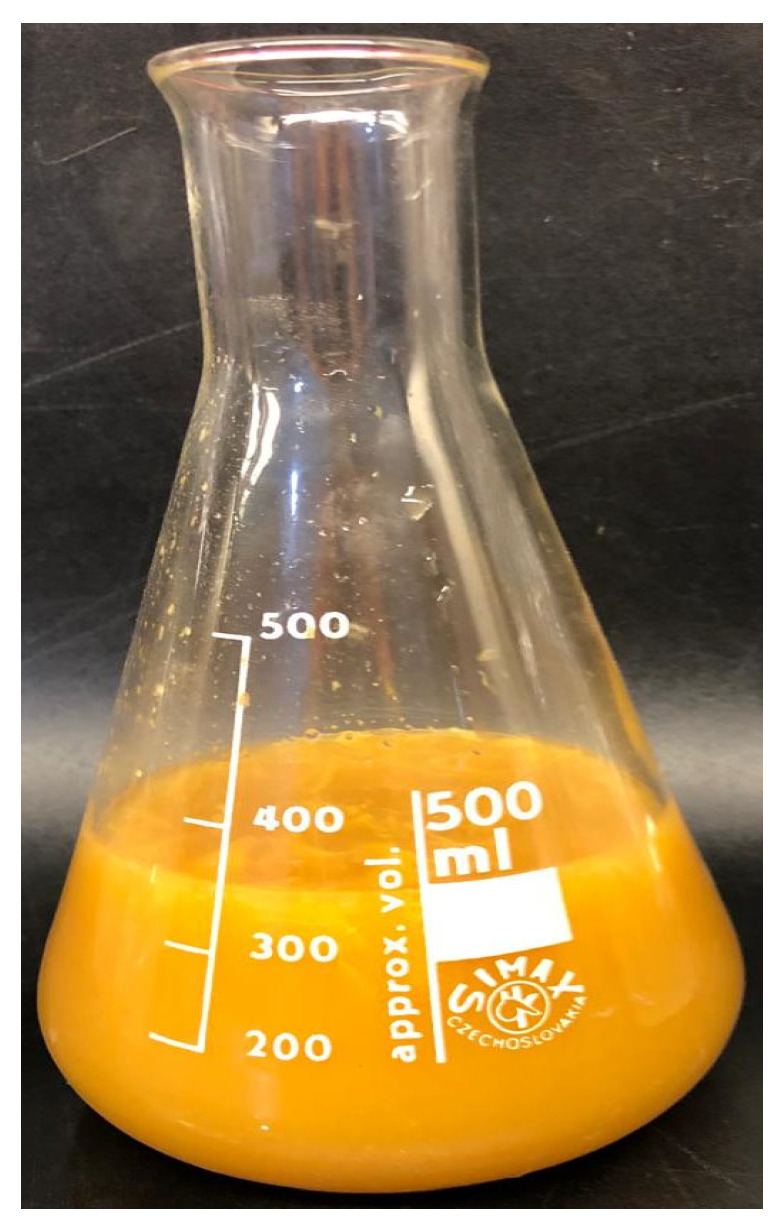
Orange pomace fermentation liquor (OPFL) is produced by the spontaneous fermentation of orange pomace.

After 15 days of incubation, the fermentation liquor exhibited a turbid, yellowish appearance with an overlay of orange fragrance, attributable to the presence of orange essential oils (Figure 2). This distinctive aroma suggests an interaction between the fermentation process and the volatile compounds from the orange peels.

### 3.2. Antimicrobial Activity Assessment

**Figure 3:**
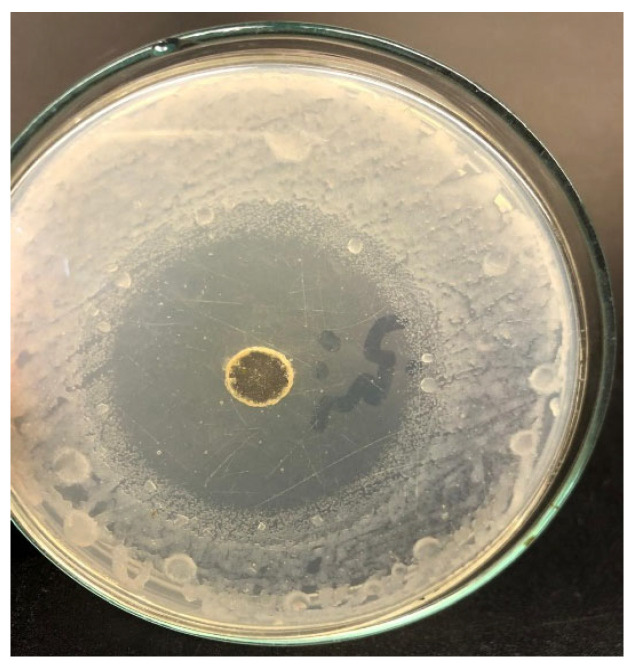
The anti-microbial properties of the orange peel fermentation liquor (OPFL) against *Pectobacterium carotovorum*—the causative agent of soft rot.

The antimicrobial efficacy of OPFL was evaluated using a well diffusion assay (Figure 3). The assay demonstrated a pronounced antimicrobial activity against *Pectobacterium carotovorum*, with the OPFL producing a clear zone of inhibition measuring 47 mm in diameter. In contrast, control wells filled with sterile water showed no inhibitory effect. These results underscore the significant antimicrobial potential of the OPFL, indicating its capability to effectively inhibit the growth of *Pectobacterium carotovorum* and suggesting its utility as a natural antimicrobial agent.

### 3.3. Identification of Bacterial Colonies in Liquor Fermentation 

The bacterial colonies identified in the orange pomace fermentation liquor exhibit characteristics consistent with the Lactobacillus genus or related lactic acid bacteria (LAB). These colonies are Gram-positive, rod-shaped, and non-motile, as indicated by Table 1.

They also display a negative catalase reaction, further supporting their classification as LAB. The microbial growth patterns observed provide additional insight into their identity. Robust growth was noted on MRS agar, a medium specifically designed to support LAB, while weak growth was observed on both nutrient agar and potato dextrose agar (Table 2). This weak growth on non-selective media is consistent with the fastidious nature of lactobacillus, which typically requires enriched media for optimal growth.

Furthermore, the fermentation liquor’s properties, including its acidic pH and high electrical conductivity, align with the acidophilic nature of the Lactobacillus species, which are known to thrive in acidic environments. The absence of coliforms and the presence of antimicrobial activity against *Pectobacterium carotovorum* suggest a controlled and beneficial microbial profile, reinforcing the likelihood that the isolated bacteria belong to the Lactobacillus genus or related LAB groups (Table 1 and Table 2).

### 3.4. Response of Sprouted Barley to Inoculation with OPFL

The interaction between orange pomace fermentation liquor inoculation and different barley varieties produced a significant positive effect on key physiological and morphological parameters, including chlorophyll content, protein percentage, and plant height (Table 3). 

#### 3.4.1. Impact of OPFL Inoculation on Chlorophyll Content

**Figure 4:**
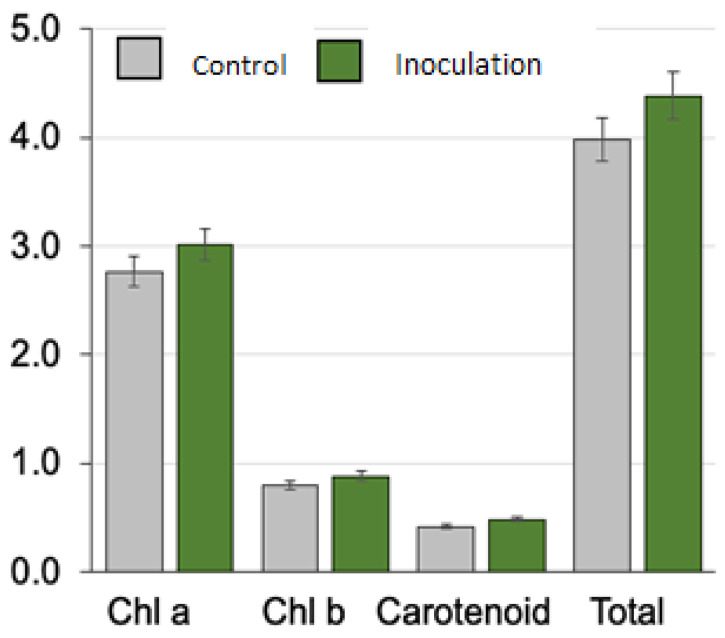
Effect of inoculation with fermentation liquor (OPEL) on barley (*Hordeum vulgare* L. var. Giza 2000) photosynthesis pigments (mg/g FW), i.e., chlorophyll a (Chl a), chlorophyll b (Chl b), carotenoid, and total chlorophyll (Total). Data are mean ± SD.

The application of OPFL significantly influenced the chlorophyll content of all barley varieties tested (Table 3 and Figure 4). Chlorophyll a (Chl a), chlorophyll b (Chl b), and total chlorophyll content were consistently higher in OPFL-inoculated plants compared to the control groups. The most pronounced increase in chlorophyll a was observed in Giza 2000, where Chl an increased from 2.94 mg/g in the control group to 3.29 mg/g in the inoculated group, representing an 11.9% increase. Similarly, total chlorophyll content increased from 4.28 mg/g to 4.74 mg/g in Giza 2000, reflecting an overall 10.7% enhancement following inoculation. In Giza 138, Chl increased from 2.70 mg/g to 2.96 mg/g while, in Giza 132, it rose from 2.88 mg/g to 2.99 mg/g after inoculation. These results indicate that the increase in chlorophyll content, while more substantial in Giza 2000, was consistent across all tested varieties. The positive effect of OPFL inoculation on chlorophyll content suggests that the fermentation liquor promotes photosynthetic efficiency, which could enhance plant growth and yield.

#### 3.4.2. Changes in Protein Content

**Figure 5:**
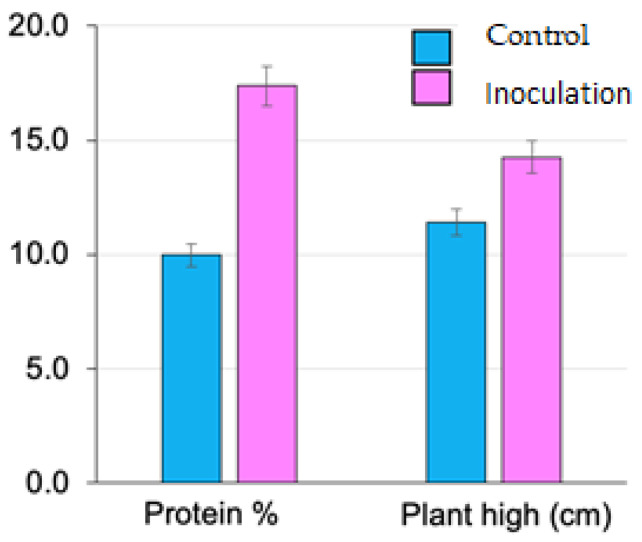
Effect of inoculation with fermentation liquor (OPEL) on height (cm) and protein content (%) of the barley plant (*Hordeum vulgare* L. var. Giza 2000). Data are mean ± SD.

Protein content exhibited a substantial increase in all inoculated barley varieties, with the most striking result observed in Giza 2000 (Table 3 and Figure 5). In Giza 2000, the protein percentage rose from 12.15% in the control to 22.07% in the OPFL-inoculated plants, representing an 81.6% increase. Similarly, in Giza 138, protein content increased from 9.48% to 17.19%, a significant 81.3% rise. Giza 2000 and Giza 126 also experienced considerable increases in protein percentage, with Giza 132 rising from 8.08% to 14.48% and Giza 126 increasing from 10.23% to 15.75%.

The dramatic rise in protein content across all varieties following inoculation suggests enhanced nitrogen assimilation in the OPFL-treated plants. These findings are particularly important for barley grown as a feed crop, as the increased protein content improves its nutritional value. Given that microbial activity, particularly from LAB in OPFL, is known to promote nitrogen cycling, it is plausible that these microbes contributed to the significant increase in protein levels [24].

#### 3.4.3. Effects on Plant Height

Plant height also increased significantly in response to OPFL inoculation across all barley varieties, with the largest increases observed in Giza 2000 and Giza 138 (Table 3 and Figure 5). In Giza 2000, plant height increased from 13.6 cm in the control group to 16.4 cm in the inoculated group, representing a 20.6% increase. Giza 138 showed a similar trend, with plant height rising from 12.0 cm to 15.6 cm, a 30.0% increase. In Giza 132, height increased from 10.4 cm to 12.2 cm, while in Giza 126, height increased from 9.6 cm to 12.8 cm after inoculation. The significant rise in plant height observed across all varieties suggests that OPFL inoculation promotes vegetative growth. The increased height may be linked to enhanced nutrient uptake, particularly nitrogen, facilitated by microbial activity in the rhizosphere. This is supported by the concurrent increases in chlorophyll content and protein percentage, both of which are associated with improved photosynthetic efficiency and nitrogen assimilation.

#### 3.4.4. Fresh Biomass Yield

**Figure 6:**
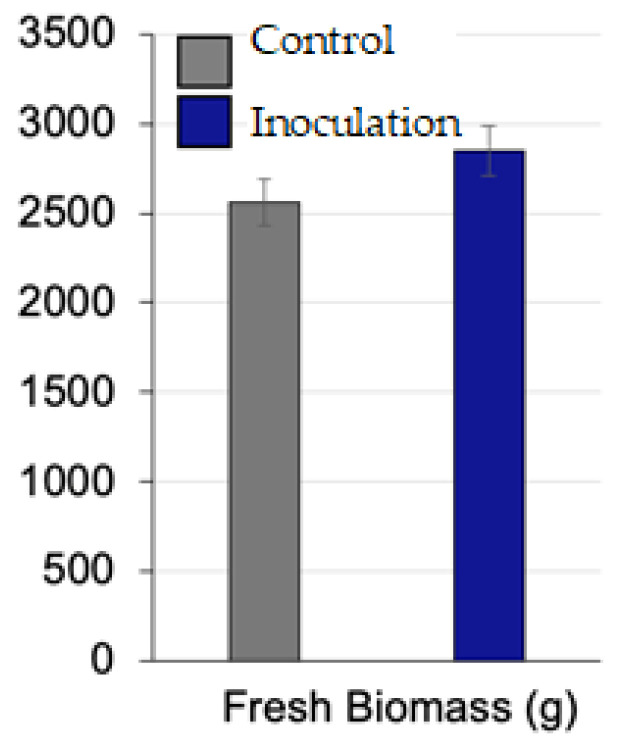
Effect of inoculation with fermentation liquor (OPEL) on barley (*Hordeum vulgare* L. var. Giza 2000) fresh biomass. Data are mean ± SD.

Despite the significant increases in chlorophyll content, protein percentage, and plant height, the effects of OPFL inoculation on fresh biomass yield were less pronounced (Table 3 and Figure 6). While biomass yield increased in all inoculated varieties, the differences between control and inoculated groups were not statistically significant. For example, Giza 2000 showed an increase in fresh biomass from 2735.0 g in the control to 2945.0 g in the inoculated group, representing a 7.7% increase. Giza 138 and Giza 132 also showed moderate increases, with biomass rising from 2299.3 g to 2626.7 g in Giza 138 and from 2375.0 g to 2756.0 g in Giza 132. Giza 126 exhibited the highest fresh biomass yield, increasing from 2826.0 g to 3065.7 g, an 8.5% increase.

While the observed increases in fresh biomass yield suggest a positive effect of OPFL inoculation, the lack of statistical significance indicates that the differences may not be solely attributable to the inoculation. It is possible that the experiment’s duration or the specific growth conditions may have influenced these results, and longer-term studies might reveal more substantial biomass gains.

### 3.5. Comparative Performance of Barley Varieties

The data clearly show that Giza 2000 consistently outperformed the other varieties across all measured parameters, particularly in chlorophyll content, protein percentage, and plant height (Table 2). Giza 2000 exhibited the highest levels of chlorophyll (3.29), total chlorophyll (4.74), protein content (22.07%), and plant height (16.4 cm) following OPFL inoculation. These findings suggest that Giza 2000 is particularly responsive to OPFL inoculation and may have a higher potential for yield and quality improvement compared to the other tested varieties. Giza 138 also showed notable improvements, with a significant rise in chlorophyll a, protein content, and plant height. Giza 132 and Giza 126, while still showing improvements, exhibited lower overall performance in terms of chlorophyll content and protein percentage compared to Giza 2000 and Giza 138. However, Giza 126 had the highest fresh biomass yield among all varieties, suggesting that it may perform well under specific conditions that favor biomass production over physiological traits, such as chlorophyll and protein content. In addition, statistical analysis confirmed the significance of the differences observed in chlorophyll content, protein percentage, and plant height between control and inoculated groups. The least significant difference (LSD) at 0.05 confidence level revealed highly significant increases in chlorophyll a, chlorophyll b, carotenoids, total chlorophyll, protein percentage, and plant height across all varieties (Table 4). However, the differences in fresh biomass yield were not statistically significant, suggesting that further studies are needed to explore the long-term effects of OPFL inoculation on biomass production. Inoculation with OPFL significantly enhanced a variety of physiological and growth parameters in barley (Table 4). For instance, chlorophyll content increased by 9.08%, from 2.76 in the control group to 3.01 in the inoculated group (LSD 0.05:0.001). Chlorophyll b rose by 10.74%, carotenoids by 16.77%, and total chlorophyll content saw a 10.21% increase. The protein percentage experienced a remarkable 74.04% increase, from 9.98% in the control to 17.37% in the inoculated group (LSD 0.05:0.19), indicating enhanced nitrogen assimilation. Plant height increased by 25.00%, while fresh biomass also improved by 11.33% with inoculation. These results underscore the efficacy of orange pomace liquor as a biostimulant, improving both the physiological and morphological traits of barley plants, with significant gains in chlorophyll content, protein levels, and plant height.

The observed increase in protein content could be attributed to the growth and activity of LAB in the rhizosphere of barley. LAB are known to thrive in nutrient-rich environments, such as those created by organic amendments like OPFL [25]. As these bacteria proliferate, they likely produce microbial protein, which can accumulate in the rhizosphere [26].

Since sprouted barley is consumed whole as feed, the presence of microbial protein from LAB may complement the natural plant proteins, leading to a noticeable increase in overall protein content. This combination of plant and microbial protein enhances the nutritional quality of the barley, making it a more valuable feed source with improved protein levels.

## 4. Discussion

The present study demonstrated the significant impact of OPFL on the physiological and growth parameters of different barley varieties. The inoculation of OPFL resulted in marked improvements in chlorophyll content, protein percentage, and plant height, particularly in the Giza 2000. These results align with the hypothesis that OPFL, rich in microbial and organic compounds, acts as an effective biostimulant, enhancing plant growth and nutritional quality.

The significant increase in chlorophyll content, particularly chlorophyll a and total chlorophyll, across all barley varieties following OPFL inoculation indicates enhanced photosynthetic efficiency. Chlorophyll is a critical component in the photosynthesis process, and its increase suggests that the inoculated plants may have improved their capacity to capture light energy, thus enhancing biomass production. Previous studies have shown that organic amendments, such as compost, biochar, and fermentation products, can increase chlorophyll content in crops by improving soil structure and nutrient availability, which facilitates better nutrient uptake by plants [27]. This improvement in chlorophyll content can be attributed to the role of OPFL in the rhizosphere, where the microbial activity of LAB and other microorganisms promotes the solubilization of nutrients, including nitrogen and phosphorus, which are vital for chlorophyll biosynthesis [6]. Moreover, LAB is known to produce plant growth-promoting substances, such as indole-3-acetic acid (IAA), which stimulates root growth, increasing nutrient and water absorption. The increased availability of nutrients in the soil, coupled with improved root architecture, is likely to have facilitated the observed rise in chlorophyll levels and overall plant vigor in inoculated barley.

The most striking result of the study was the significant increase in protein percentage, particularly in Giza 2000, where the protein content rose from 12.15% in the control group to 22.07% in the inoculated group. This substantial increase is indicative of enhanced nitrogen assimilation, which could be driven by the microbial activity in the rhizosphere. LAB, which thrives in the presence of organic matter, such as orange pomace, can contribute to the production of microbial protein. This microbial protein, combined with the plant’s own protein, may explain the notable increase in total protein levels in the inoculated plants. The role of microbial protein in increasing the overall protein content in feed crops, such as barley, is well documented. Several studies have reported that the incorporation of organic amendments rich in microbial biomass can enhance the protein content of plants, particularly when LAB and other beneficial microbes are involved [1,2,11]. These bacteria can fix nitrogen, making it more readily available for plant uptake, while also producing amino acids that may directly contribute to the protein synthesis in plants. In the context of barley grown for feed, the higher protein content significantly boosts its nutritional value, offering a dual benefit from both plant and microbial sources of protein.

Inoculation with OPFL also led to a significant increase in plant height, particularly in Giza 2000, which showed a 25% increase compared to the control. While the fresh biomass yield showed no statistically significant differences, the increased plant height suggests that OPFL contributed to overall plant vigor. The lack of significant difference in fresh biomass yield may be due to the fact that the experiment focused on early growth stages, where height is more responsive to nutrient availability than overall biomass accumulation. Previous studies have demonstrated that organic amendments can increase plant height by improving soil health, nutrient availability, and microbial diversity in the rhizosphere [27]. The enhanced microbial activity in the OPFL-treated soils likely facilitated better nutrient absorption, particularly nitrogen, which is essential for vegetative growth. The absence of significant biomass differences may also be due to the relatively short duration of the experiment, suggesting that longer-term studies are needed to evaluate the full impact of OPFL on biomass accumulation.

The positive effects of OPFL on barley growth can largely be attributed to the microbial interactions in the rhizosphere. LAB, which were detected in the OPFL-treated soils, are known to promote plant growth through a variety of mechanisms, including phosphate solubilization, nitrogen fixation, and the production of phytohormones, such as IAA [8]. These processes enhance nutrient availability, improving plant health and resilience to environmental stressors. Additionally, the antimicrobial potential of OPFL, particularly its ability to suppress *Pectobacterium carotovorum*, suggests that it may also play a role in protecting plants from pathogens. This dual role of promoting growth and protecting plants from disease positions OPFL as a promising organic amendment for sustainable agriculture. The suppression of pathogens by LAB has been widely reported in the literature, with these bacteria producing organic acids and bacteriocins that inhibit the growth of harmful microbes [28].

While the current study demonstrates the potential of OPFL as a biostimulant, several avenues for future research remain. First, the long-term effects of OPFL on barley growth and yield should be explored, particularly during the later stages of development. The lack of significant differences in fresh biomass suggests that OPFL may have a more pronounced effect on physiological parameters, such as chlorophyll and protein content, than on overall biomass in the early stages of growth. Further research should also investigate the specific microbial communities that develop in OPFL-treated soils and their functional roles in promoting plant growth. The exact mechanisms through which LAB and other beneficial microbes influence nutrient cycling and plant growth are still not fully understood and could be a key area of study. Additionally, research could explore the potential of OPFL to enhance the growth of other crops, particularly those with similar nitrogen and nutrient demands as barley. Finally, the broader agricultural implications of using OPFL should be considered, including its potential as a sustainable, low-cost alternative to chemical fertilizers. As the demand for environmentally friendly agricultural practices increases, OPFL could be an innovative solution for enhancing crop quality and yield while promoting soil health and reducing reliance on synthetic inputs.

The findings of this study could have significant implications for the development of cost-effective and environmentally friendly strategies for improving crop yield and quality, particularly in the context of animal feed production. Moreover, the research highlights the broader potential of utilizing agricultural byproducts, such as citrus pomace, to promote ecological farming methods and reduce reliance on chemical inputs. As the agricultural sector faces increasing pressure to produce more food sustainably, the use of fermented byproducts, like OPFL, may offer a practical and scalable solution to improve both crop productivity and environmental stewardship.

## 5. Conclusions

The application of orange pomace fermentation liquor significantly improved chlorophyll content, protein percentage, and plant height in barley, with notable effects in the Giza 2000 variety. These enhancements are attributed to the active microbial content in OPFL, which is likely to have promoted nitrogen assimilation and photosynthetic efficiency. Although the increase in fresh biomass was less pronounced, OPFL shows promise as a sustainable bio-fertilizer that reduces reliance on synthetic inputs while boosting the nutritional quality of barley as animal feed. Further research is warranted to explore long-term effects on yield and the potential broader application of OPFL in sustainable agriculture.

## Figures and Tables

**Table 1:** Characterization of bacterial colonies detected in the orange pomace fermentation liquor (OPFL).

Parameter	Value
Shape	Rod shaped
Motility	Non motile
Spore formation	Non motile
Catalase reaction	Negative

**Table 2:** Characters of liquor derived after spontaneous fermentation of orange pomace.

Parameter	Value
Appearance	Yellowish turbid liquid
Smell	Orange scent competes with fermentation smell
pH	4.3 ± 0.05
EC (dS/m)	9.3 ± 0.23
Total acidity (g acetic acid/L)	2 ± 0.01
Total microbial count on MRS	5 × 10^7^ ± 0.02
Growth on nutrient agar	Weak growth was observed after incubation for 7 days
Growth on potato dextrose agar (PDA)	Weak growth was observed after incubation for 7 days
Detection of coliform	Not detected
Blood hemolysis	No blood hemolysis
Antimicrobial potential	Active against *Pectobacterium carotovorum*
IAA	Present
Phosphate solubilization potential	Positive

**Table 3:** Effect of orange pomace liquor (OPEL) inoculation on various parameters of different barley varieties (*Hordeum vulgare* L.).

Interaction	Chl a mg/g	Chl b mg/g	Total Chl mg/g	Carotenoid mg/g	Protein %	Plant Height cm	Fresh Biomass g
V1-con.	2.94 ± 0.02	0.974 ± 0.01	4.28 ± 0.04	0.37 ± 0.01	12.15 ± 0.11	13.6 ± 0.19	2735 ± 30
V1-in.	3.29 ± 0.02	0.967 ± 0.03	4.74 ± 0.03	0.48 ± 0.02	22.07 ± 0.19	16.4 ± 0.15	2945 ± 58
V2-con.	2.70 ± 0.02	0.832 ± 0.04	4.02 ± 0.02	0.49 ± 0.03	9.48 ± 0.16	12.0 ± 0.20	2299 ± 59
V2-in.	2.96 ± 0.01	0.954 ± 0.002	4.43 ± 0.01	0.51 ± 0.01	17.19 ± 0.12	15.6 ± 0.24	2627 ± 55
V3-con.	2.88 ± 0.01	0.734 ± 0.02	4.03 ± 0.01	0.42 ± 0.02	8.08 ± 0.10	10.4 ± 0.21	2375 ± 46
V3-in.	2.99 ± 0.03	0.827 ± 0.00	4.31 ± 0.01	0.49 ± 0.01	14.48 ± 0.15	12.2 ± 0.23	2756 ± 42
V4-con.	2.54 ± 0.01	0.648 ± 0.01	3.58 ± 0.02	0.40 ± 0.00	10.23 ± 0.11	9.6 ± 0.11	2826 ± 35
V4-in.	2.81 ± 0.02	0.782 ± 0.01	4.06 ± 0.01	0.46 ± 0.01	15.75 ± 0.12	12.8 ± 0.21	3066±
L.S.D. 0.05	0.002 **	0.003 **	0.02 **	0.005 **	0.233 **	0.199 **	n.s.

V1; Giza2000, V2; Giza 138, V3; Giza 132; V4; Giza 126. ** significant at 0.01 level; n.s., not significant.

**Table 4:** Effect of different barley (*Hordeum vulgare* L.) varieties on quality and biomass yield parameters.

Varieties	Chl a mg/g	Chl b mg/g	Total Chl mg/g	Car. mg/g	Protein %	Plant Height cm	Fresh Biomass g
Giza 2000	3.11 ± 0.02	0.97 ± 0.02	4.51 ± 0.03	0.43 ± 0.01	17.11 ± 0.02	15.00 ± 0.26	2840 ± 33
Giza 138	2.83 ± 0.03	0.89 ± 0.01	4.23 ± 0.01	0.50 ± 0.02	13.33 ± 0.04	13.80 ± 0.35	2463 ± 35
Giza 132	2.94 ± 0.03	0.78 ± 0.01	4.17 ± 0.02	0.45 ± 0.00	11.28 ± 0.05	11.30 ± 0.24	2566 ± 42
Giza 126	2.68 ± 0.02	0.72 ± 0.01	3.82 ± 0.02	0.43 ± 0.01	12.99 ± 0.02	11.20 ± 0.12	2946 ± 51
L.S.D. 0.05	0.005 **	0.009 **	0.02 **	0.003 **	0.269 **	0.245 **	77.2 **

** significant at 0.01 level.

## Data Availability

Data will be made available upon the request.
